# Impact of Wellness4Teachers Supportive Text Messaging Program in Reducing Psychological Symptoms among Teachers: Longitudinal and Naturalistic Controlled Evaluation

**DOI:** 10.1192/j.eurpsy.2026.11362

**Published:** 2026-07-31

**Authors:** B. Agyapong, Y. Wei, P. Brett-MacLean, V. I. O. Agyapong

**Affiliations:** 1 Psychiatry, University of Alberta, Edmonton; 2 Psychiatry, Dalhousie University, Hlifax, Canada

## Abstract

**Introduction:**

The Wellness4Teachers program, a daily supportive text messaging program, was implemented to help mitigate psychological issues among teachers in Canada.

**Objectives:**

To assess whether Wellness4Teachers can reduce the severity and prevalence of psychological issues and improve resilience among Alberta, Newfoundland and Labrador, and Nova Scotia teachers.

**Methods:**

This study used both longitudinal and naturalistic controlled trial designs. For the longitudinal study, Wellness4Teachers subscribers completed surveys at baseline, six weeks, three months, and six months. The naturalistic controlled trial compared two groups of Wellness4Teachers subscribers: the intervention group (IG), consisting of subscribers who received once-daily supportive text messages for six months and completed sixth-month evaluation between March 1 and June 30, 2023, and the control group (CG), composed of teachers who joined Wellness4Teachers during the same period and only completed the baseline survey but had not yet received any text messages.

**Results:**

For the longitudinal study, the mean scores on the Perceived Stress Scale (PSS-10), the Patient Health Questionnaire (PHQ-9), the 3rd and 9th question of the PHQ-9, and the Generalized Anxiety Disorder (GAD-7) were significantly lower by 5.5%, 18.58%, 17.54%, 37.5% and 13.52% at six months compared to baseline respectively for subscribers who completed the baseline survey and at least one follow-up survey. Additionally, the prevalence of likely Moderate Depressive Disorder (MDD) was significantly lower by 14.2% at six months compared to the baseline for subscribers who completed the baseline and at least one follow-up survey. In the naturalistic controlled trial, the mean scores on the PSS-10, the PHQ-9, and the GAD-7 were significantly lower in the IG by 6.04%, 21.16%, and 22.48% compared to the CG. In addition, the prevalence of likely MDD and GAD were significantly lower by 20.6% and 20.3%, respectively, for the IG compared to the CG. Finally, participants in the CG were 2.37, 2.13, and 2.74 times more likely to meet the cutoff threshold for high Stress, likely MDD, and likely GAD than participants in the IG.

**Conclusions:**

The Wellness4Teachers program may be an effective intervention for reducing psychological symptoms among teachers. The Wellness4Teachers program appears to be a promising intervention for alleviating psychological symptoms among teachers.

**Disclosure of Interest:**

None Declared

